# Video analysis of the escape flight of Pileated Woodpecker *Dryocopus pileatus*: does the Ivory-billed Woodpecker *Campephilus principalis *persist in continental North America?

**DOI:** 10.1186/1741-7007-5-8

**Published:** 2007-03-15

**Authors:** J Martin Collinson

**Affiliations:** 1 University of Aberdeen, School of Medical Sciences, Institute of Medical Sciences, Foresterhill, Aberdeen AB25 2ZD, UK

## Abstract

**Background:**

The apparent rediscovery of the Ivory-billed Woodpecker *Campephilus principalis *in Arkansas, USA, previously feared extinct, was supported by video evidence of a single bird in flight (Fitzpatrick *et al*, *Science *2005, **308**:1460–1462). Plumage patterns and wingbeat frequency of the putative Ivory-billed Woodpecker were said to be incompatible with the only possible confusion species native to the area, the Pileated Woodpecker *Dryocopus pileatus*.

**Results:**

New video analysis of Pileated Woodpeckers in escape flights comparable to that of the putative Ivory-billed Woodpecker filmed in Arkansas shows that Pileated Woodpeckers can display a wingbeat frequency equivalent to that of the Arkansas bird during escape flight. The critical frames from the Arkansas video that were used to identify the bird as an Ivory-billed Woodpecker are shown to be equally, or more, compatible with the Pileated Woodpecker.

**Conclusion:**

The identification of the bird filmed in Arkansas in April 2004 as an Ivory-billed Woodpecker is best regarded as unsafe. The similarities between the Arkansas bird and known Pileated Woodpeckers suggest that it was most likely a Pileated Woodpecker.

## Background

The reported rediscovery of the Ivory-billed Woodpecker in 2004–5 in the Big Woods of Arkansas gave new impetus to efforts to conserve the mature bottomland woodlands of the south-eastern USA. Several sightings have been reported without photographic evidence being obtained [1]. Unless sightings are, however, independently verifiable on the basis of photographic or other recorded evidence, the possibility that mistakes have been made cannot be eliminated. Crucial to the scientific case for the persistence of the Ivory-billed Woodpecker was a 4 s video of a large woodpecker in flight recorded by M.D. Luneau on 25 April 2004 (henceforth referred to as the 'Luneau video') and published in 2005 [1], which was claimed to be inconsistent with the plumage patterns of the superficially similar Pileated Woodpecker (a common resident bird of the area). Both species are large, black-and-white woodpeckers [2]. The upperwing of the Ivory-billed Woodpecker is black, with white secondary feathers and white on some inner primary feathers. Pileated Woodpeckers have a largely black upperwing, with white restricted to the 'wrist' due to white bases to the primary feathers. The underwing of Pileated Woodpecker has all-white underwing coverts, giving an appearance of a white underwing with a broad black outline (the black flight feathers). These plumage differences result in the Ivory-billed Woodpecker having a *white *trailing edge to the wings (upper and lower sides), whereas the Pileated Woodpecker has a *black *trailing edge to the wings. Both species have black wing-tips. These and other plumage characteristics are shown in [1,2]. The wingbeat frequency of the bird in the Luneau video was measured at 8.6 beats s^-1^, similar to that inferred from archival sound recording of a single Ivory-billed Woodpecker, but claimed to be outside the range of Pileated Woodpeckers (which generally have slower wingbeats) [1,3].

Sibley *et al *[4] questioned the video evidence, in particular providing alternative explanations for the plumage patterns of the Luneau bird in flight and at rest. They pointed out individual frames of the Luneau video that appear to show three features that are each inconsistent with Ivory-billed Woodpecker: (1) apparently black secondary feathers on the upper surface of the left wing, (2) particularly bright white primary bases, and (3) a black band curving smoothly round the wing tip (see Figure 3 in [4]). They hypothesized that flexing of a Pileated Woodpecker's wings during flight could produce the appearance of white trailing edges on both wings in low-quality videos [4]. They offered, however, no direct evidence to show that this could cause a video of a Pileated Woodpecker to look like the bird in the Luneau video. Fitzpatrick *et al *[5] in turn rebutted some aspects of the hypothesis of Sibley *et al *[4], publishing video stills of Pileated Woodpeckers, and a model of a Pileated Woodpecker, that appeared to show a black trailing edge to the wings inconsistent with Ivory-billed Woodpecker and the Luneau video. Fitzpatrick *et al *[5] neither rebutted nor discussed the three key inconsistencies described above. Without further evidence, this became largely a theoretical debate over interpretation of field characters that were barely visible in the very small images originally obtained. On one hand, as pointed out in Sibley *et al *[4], some of the frames of the bird in the Luneau video do appear to be inconsistent with Ivory-billed Woodpecker. On the other hand, the flight pattern of the bird in the Luneau video is asserted to be atypical for Pileated Woodpecker (but matching anecdotal descriptions of Ivory-billed Woodpecker). Furthermore, the general impression of the bird in the Luneau video was that there is far too much white in the wings for it to be a Pileated Woodpecker, and that if it was a Pileated, then it must be an aberrant one with abnormally extensive white plumage. Such birds occasionally occur, and have been observed in the Arkansas study area [6].

This study was undertaken to determine whether the flight and plumage of the bird in the Luneau video really was inconsistent with either a normal or partial albino Pileated Woodpecker. Independent analyses of the plumage patterns and wingbeat frequencies observable in Pileated Woodpeckers are presented, and it is concluded that the identification of the bird in the Luneau video as definite Ivory-billed Woodpecker is probably unsafe.

## Results

On January 28 and February 5, 2006, David Nolin (DN) video-recorded Pileated Woodpeckers *Dryocopus pileatus *at a bird-feeder in Dayton, Ohio, USA. A Hi-8 Sony Handycam was used, hand-held, at approximately 5 m from the feeder. Birds on the tree trunk were alarmed by movement, and their escape flights recorded. Four escape flights were captured that approximate to that recorded for the putative Ivory-billed Woodpecker *Campephilus principalis *by Luneau in April 2004 and published in Fitzpatrick *et al *[1]. The videos are not directly equivalent because the Pileated Woodpeckers made only short escape flights to nearby trees, whereas the putative Ivory-billed Woodpecker in the Luneau video showed little sign of coming to rest before being lost from view. Nevertheless, interesting comparisons can be made.

### Wingbeat frequency of Pileated Woodpecker

The woodpecker in the Luneau video maintains a steady rapid wingbeat rate of 8.6 beats s^-1 ^for at least 8 wingbeats [1], a figure that was confirmed by independent analysis during preparation of this paper. The Pileated woodpeckers in DN's video do not do this – after initial rapid flapping immediately after take-off, they settle into a more relaxed level flight. As shown in Tables 1 and 2, although the mean wingbeat frequencies of the Pileated Woodpeckers in DN's video are slower than the 8.6 s^-1 ^recorded for the bird in the Luneau video [1,3,5] the first four wingbeats, the initial escape response, are faster than those claimed for Pileated Woodpeckers in the literature [1,3,5]. For the four escape flights, the mean frequency values for the first four wingbeats are 7.1, 6.7, 8.6, and 8.0 s^-1^, respectively. The 8.6 beats s^-1 ^of the bird identified in the Luneau video, while consistent with the limited data (n = 1; see Discussion) for Ivory-billed Woodpecker, is equally consistent with Pileated Woodpecker in its initial escape flight. The bird in the Luneau video maintains a frequency of 8.6 s^-1 ^for the next four wingbeats too, whereas the Pileated Woodpeckers recorded here all slowed their flight as they prepared to land in nearby trees. There are no data to suggest whether Pileated Woodpeckers can maintain a wingbeat frequency approaching 8.6 s^-1 ^for eight or more wingbeats, like the bird in the Luneau video. It remains possible that the flight pattern of the bird in the Luneau video is unusual for Pileated Woodpecker, but a frequency of 8.6 s^-1 ^is consistent with a Pileated Woodpecker gaining initial speed and height in escape flight, and by itself cannot be taken as strong evidence that the Luneau video bird was an Ivory-billed Woodpecker. This is discussed further below.

### Plumage pattern of Pileated Woodpeckers in flight

**Figure 1:**
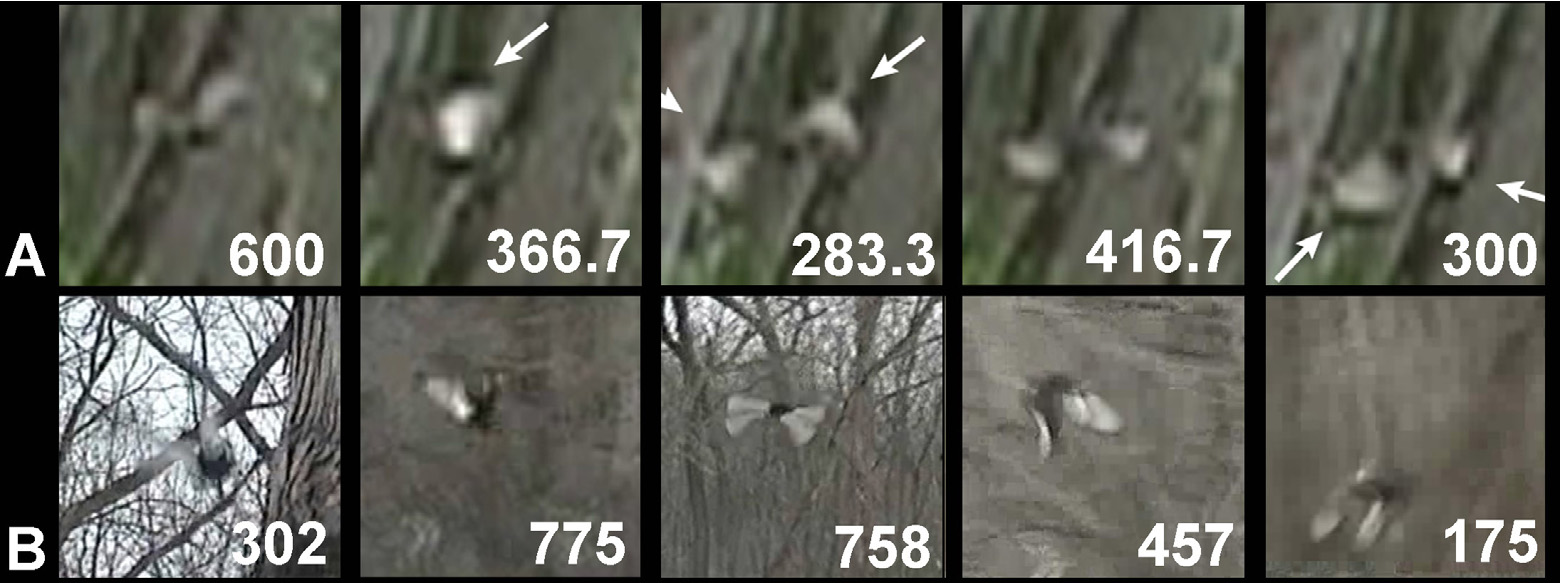
**Comparison of selected frames from the video presented in Fitzpatrick *et al *[1] of a putative Ivory-billed Woodpecker from the 'Luneau video' (Top row, A), and selected frames from David Nolin's video of known Pileated Woodpecker (Bottom row, B)**. Numbers in A identify the frames presented in [1]. In B, represent the decompiled frame numbers from DN's video. Frames chosen in A are representative of each phase of the Luneau bird's flight action, on the upstroke rising directly away from the observer (600), at the top of the upstroke flying obliquely away from the observer (366.7), at the midpoint of the downstroke flying directly away from the observer (283.2), towards (416.7) and at (300) the bottom of the downstroke. Each phase of the wingbeat cycle is consistent with frames of known Pileated Woodpeckers presented in B. There are striking similarities. The images confirm the hypothesis that the impression of extensive black wingtips recorded for the bird in the Luneau video (highlighted by white arrows in the original published images) was in fact produced by the extensive black trailing edge of a Pileated Woodpecker. It seems possible that the black trailing edge to the wing of Pileated Woodpecker was often visible in many frames of the Luneau video, but it was misinterpreted as black wingtips. The flicker and extra wings in B, frame 758 are artifacts of the impossibility of deinterlacing the video, but each wing image is clearly separable. Reproduced from [1] with permission from David Luneau.

**Figure 2:**
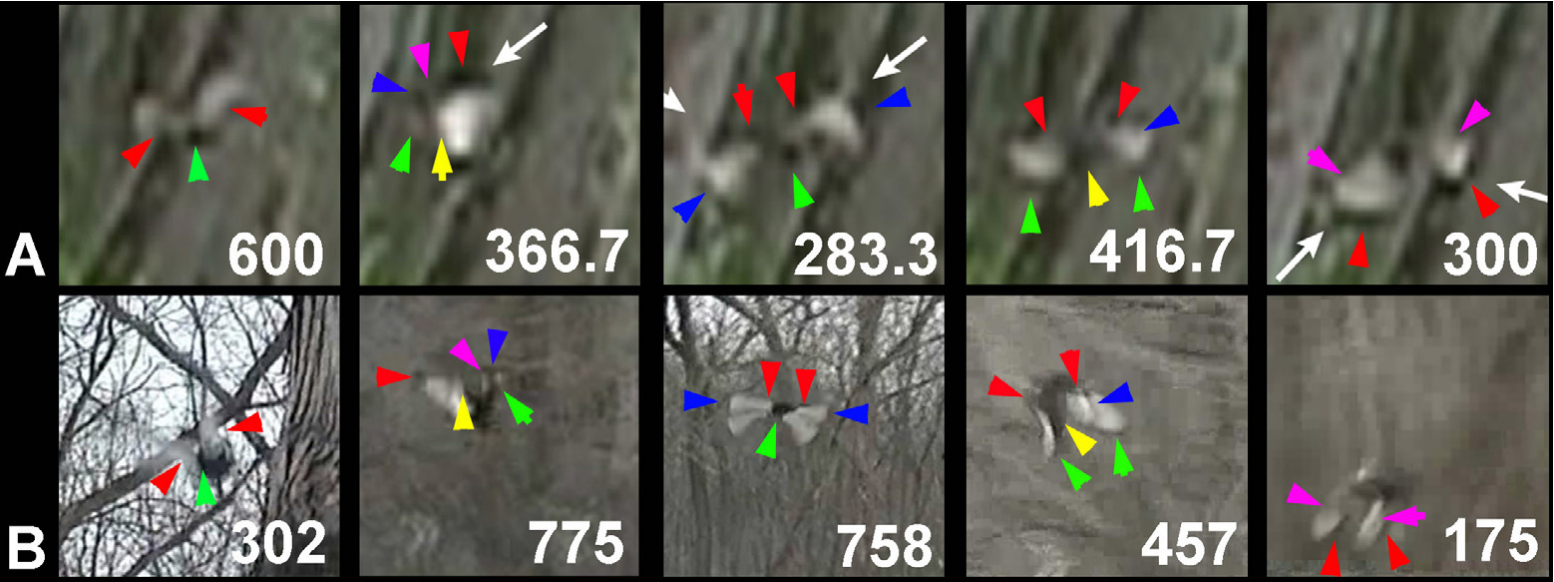
**A copy of Figure 1 with plumage similarities between comparable frames highlighted**. (A frame 600 and B 302): green arrowhead, black body with white of opposite wings apparently almost meeting; red arrowheads, apparent white trailing edge to wings. (A frame 366.7 and B 775): note these images are in reverse orientation with respect to each other because the bird in A is flying away to the right, whereas that in B is flying away to the left. Yellow arrowheads, inconspicuous trace of black trailing edge to underwing; Red arrowheads, apparent black wingtips; green arrowhead, apparent white trailing edge to upperwing; blue arrowhead, black upperwing with apparent white leading edge (purple arrowhead). (A frame 283.3 and B 758): blue arrowheads, black wingtips on apparently largely white wings; red arrowheads, apparent black leading edge to upperwings; green arrowheads, narrow, apparently all black body. (A frame 416.7 and B 457): yellow arrowheads, all black body (no white); green arrowheads, apparent black wingtips, in fact black trailing edges; red arrowheads, apparent black leading edges to wings; blue arrowheads, apparent smudges on white surface of wing. (A frame 300 and B 175): red arrowheads, apparent black wingtips. In the upper panel (the putative Ivory-billed Woodpecker), although partly obscured by a tree branch the black appears to wrap around a large panel of white in the wing (see also frame 416.7). This is difficult to reconcile with the plumage of Ivory-billed Woodpecker, except as an artifact of rapid motion, but in the lower panels is clearly identifiable as consistent with the black wingtips and trailing edge surrounding the white panel on the underwing of Pileated Woodpecker. Purple arrowheads, largely white underwing white wing surface with dusky markings, a pattern which can be characteristic of Pileated Woodpecker due to the fact that the inner wing is not translucent because of the backing of bone and other thick tissues. The white arrows were superimposed on the original figures in Fitzpatrick *et al *[1]. Reproduced from [1] with permission from David Luneau.

**Figure 3:**
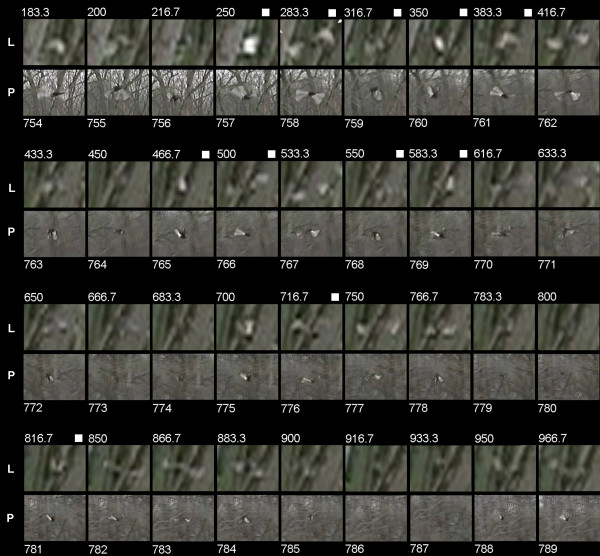
**Direct comparison of 36 frames from the Luneau video (selected frames in order from 183.3 ms to 966.7 ms; top line of each row) with equivalent frames from the fourth flight of Pileated Woodpecker in David Nolin's video (all of frames 754–789, bottom line of each row)**. The fourth escape flight from David Nolin's video was chosen because it most closely matches the trajectory of the bird in the Luneau video. Luneau data was presented at twice the frame rate of the Nolin video (which was a standard 29.97 frames s^-1^), so not all Luneau frames are shown here. The frames in each case start at the midpoint of the second wingbeat after take-off, and continue to almost the midpoint of the ninth wingbeat. Because after the fourth wingbeat, the Pileated Woodpecker in the Nolin video slows its rate of flapping, the two comparisons are not to the same timescale (Pileated sequence = 1.08 s, Luneau sequence = 0.78 s). White squares at the top of the Luneau video sequence indicate an omitted frame. The Pileated Woodpecker flies consistently obliquely away to the left, whereas the Luneau bird initially is flying obliquely away to the right. For every field, in sequence, of the Nolin Pileated video, an equivalent frame, in sequence, of the bird in the Luneau video is available. In most frames, the similarities are striking, and in no case is there a clear plumage feature on the bird in the Luneau video that is incompatible with the known Pileated Woodpecker. In contrast, note frame 350 of the Luneau video, which unambiguously shows the wing patterns of a Pileated Woodpecker. Reproduced from [1] with permission from David Luneau.

The video of Pileated Woodpeckers in flight was obtained in avi format, decompiled and examined frame by frame. Comparisons of Pileated Woodpecker images with key images of Luneau video are shown in Figures 1 and 2, and suggest a genuine resemblance between the bird in the Luneau video and a Pileated Woodpecker. Analysis is complicated by the different digital processing of the two videos, and in the case of the Nolin videos it is important to concentrate only on those frames or part-frames where apparent plumage features are not an artifact of blurred images. Thirty-six frames from the fourth example of Pileated escape flight, which most resembled the flight path of the Luneau video bird, were analysed systematically frame by frame. They represent seven complete wingbeats (1.20 s from the middle of the second wingbeat to middle of wingbeat 9) and were directly compared frame-by-frame with the equivalent fields (middle wingbeat 2 – middle wingbeat 9) of the Luneau video. This comparison is shown in Figure 3. The images of the birds are not identical, but in every frame of the 36 frames available, there are sufficient similarities to suggest that the bird in the Luneau video is consistent with the known Pileated Woodpecker. Further comparisons of the Luneau bird with the other three Pileated escape flights recorded are presented in the supplementary material (see Additional file 1).

Key findings of the video analysis are:

1. Pileated Woodpeckers flying near-horizontally away from the observer show much more white in poor-quality video than would be expected from their general plumage pattern. They present an appearance of a black-bodied bird with largely white wings and black wingtips, very similar to the bird in the Luneau video; compare in particular Figure 1B, frame 758, with Figure 1A, frame 283.3. The expected appearance of the upperwing of Pileated Woodpecker – mostly black with a small white patch at the base of the primaries – is often not seen, and is only clearly resolvable when birds are flying near-vertically before landing on a tree trunk; something the bird in the Luneau video did not do.

2. The black trailing edge to the underwing of Pileated Woodpecker is often very inconspicuous and may disappear completely. Due to motion and flexion of the wing, the black trailing edge is much more obvious towards the wingtips. This produces an apparent plumage pattern that matches the patterns shown by the Luneau video bird (compare Figure 1B, frames 175 and 457 with Figure 1A, frames 300 and 416.7). In many frames of Pileated Woodpecker, a black trailing edge to the wing is discernable (though due to bleeding of white as a video artifact, it appears narrower than it really is). However, analysis of the bird in the Luneau video in light of images of known Pileated Woodpeckers confirms that a similar black trailing edge to the wing is discernable in some frames of the Luneau video (compare Figure 1B, frame 775 with Figure 1A, frame 366.7: the apparent plumage patterns are similar, and inconsistent with Ivory-billed Woodpecker). It is argued here that the hypothesis put forward in Sibley *et al *[4] is correct, and that the black trailing edge of the underwing of Pileated Woodpecker can indeed, due to flexion of the wings during the downstroke, be misinterpreted as the black leading edge and wingtips of the upperwing of an Ivory-billed Woodpecker.

3. Figure 3 shows that the plumage patterns shown by the Luneau bird, throughout several wingbeat cycles, are compatible with Pileated Woodpecker. The three plumage features described in Sibley *et al *[4] that are incompatible with Ivory-billed Woodpecker (black secondary feathers on upper surface of left wing, brighter white primary bases, and a black band curling round the wing tip) are seen consistently in the Luneau video and are recapitulated throughout the video of Pileated Woodpecker.

## Discussion

Evidence is presented here to show that the distinctive plumage features of Pileated Woodpecker are surprisingly difficult to resolve in poor-quality video of birds in escape flight away from the camera, and that they can show apparent plumage patterns that might more readily be associated with Ivory-billed Woodpecker. Irrespective of the identity of the bird in the Luneau video, this knowledge will be critical to assessment of further claims of Ivory-billed Woodpeckers during the current intensive search effort. It is, however, suggested here that critical frames used for identification of the Luneau video woodpecker as an Ivory-billed Woodpecker are also consistent with Pileated Woodpecker. The wingbeat frequency of the bird in the Luneau video is also perhaps consistent with Pileated Woodpecker, at least for short periods of flight.

Analysis of the videos of Pileated Woodpecker has supported the hypothesised interpretations of key frames of the Luneau video by Sibley *et al *[4]. Although the rebuttal of that comment in Fitzpatrick *et al *[5] asserted that flexion and motion of wings of Pileated Woodpeckers could not produce the images seen in the Luneau video, it has been shown here that they can.

The Luneau video as presented in Fitzpatrick *et al *[1], shows features that are consistent with Pileated Woodpecker, and inconsistent with Ivory-billed Woodpecker. It is argued in this paper that, in fact, the black trailing edge of the wing of a Pileated Woodpecker is seen clearly in the Luneau video, during the downstroke of the wingbeat cycle, but that it has been misinterpreted as black wingtips (Figure 1, 2, 3).

A fuller analysis of the Luneau video by the Cornell University team is presented online [7]. Although it is not peer-reviewed, the points this article makes should be taken into account. The authors summarise nine diagnostic traits from their analysis of the Luneau video that identify the bird as Ivory-billed Woodpecker. These are listed and discussed point-by point below.

1. 'The underwing pattern in flight consistently appears largely white, giving the appearance of having black wingtips but lacking any black along the rear, or trailing edge.'

Data presented in this paper show that this statement is not wholly supported, and in any case the underwing of Pileated Woodpeckers can present the same appearance.

2. 'The upperwing pattern in flight consistently shows a broad, white trailing edge, with no frames demonstrating the conspicuous dark rear border to be expected of normal Pileated Woodpeckers.'

Notwithstanding that certain frames of the Luneau video (e.g. frame 350) *do *appear to show a black trailing edge to the upperwing, data presented in this paper shows that, at this angle of view and resolution of video, Pileated Woodpeckers also may fail to show this feature. This analysis has shown that the hypothesis presented in Sibley *et al *[4] is plausible, i.e. that some of the frames interpreted by [1] to show the upperwing of an Ivory-billed Woodpecker may in fact show large amounts of white and the black trailing edge from the underwing of a Pileated Woodpecker.

3. 'The wings are longer relative to the body diameter than in Pileated Woodpecker and consistent with the wing shape of Ivory-billed Woodpecker.'

Fitzpatrick *et al *[5] agreed that accurate measurements were not possible from the video images presented in their original paper [1], and it seems unlikely that much confidence can be placed in the wing-length measurements of the bird in the Luneau video. Comparison of, for example, Figure 1A, frame 283.3 with Figure 1B, frame 578 suggests that any differences will be very difficult to prove.

4. 'Reenactment of the scene using life-sized, realistically painted, dynamically flapping models produced images remarkably similar to those of the Luneau video using the Ivory-billed Woodpecker model, and images clearly identifiable as Pileated Woodpecker using a model of that species.'

Interpretation of model re-enactments is hampered by the fact that the stiff, flat-winged models cannot reflect the wing flexion and curvature of real birds. Reenactment of the scene using real Pileated Woodpeckers has produced images remarkably similar to the Luneau video.

5. 'The wingbeat frequency is 8.6 beats per second, which is almost identical to that recorded for Ivory-billed Woodpecker (as documented by one acoustic record from 1935). The wing-beat frequencies of Pileated Woodpecker are not known to exceed 7.5 beats per second, and more typically range between 3 and 6 beats per second.'

The fact that in only four recorded escape flights of Pileated Woodpecker, two were recorded for which the initial escape flight wingbeat frequency (8.0 s^-1 ^and 8.6 s^-1^) exceeded that previously recorded for this species shows that previous datasets were too limited to make this conclusion. Birds flap more rapidly at take off to gain altitude and speed than they do in sustained level flight: Pileated Woodpecker flight data in the literature [1,4,5] was derived from the work of Tobalske [8], which explicitly excluded the initial take-off period, and therefore cannot be used to support the elimination of Pileated Woodpecker in the Luneau video. Furthermore, the bird in the Luneau video is consistently gaining height from a low position above water and, whatever its species, might be expected to flap more rapidly than if it were in level flight. Tanner [9] noted that Pileated Woodpeckers can maintain extended fast direct flight. He was of the opinion that flight pattern was not a useful character for separating the two species in the field, and that Pileated Woodpeckers frequently fly in a manner that was in no way different to Ivory-billed Woodpeckers.

The figure of 8.6 wingbeats per second for the Luneau bird (data reanalysed here) is taken as consistent with Ivory-billed Woodpecker on the basis of analysis of a single archival audio recording [3]. The Ivory-billed Woodpecker in that audio tape is clearly flapping its wings, but without accompanying visual confirmation it is not clear that it is in flight. In general, larger birds are expected to flap their wings more slowly than smaller birds of comparable wing morphology. Tobalske [8] showed that, across species, smaller woodpeckers tend to flap more quickly than larger ones, and that there was considerable intraspecific variation. The assertion that Ivory-billed Woodpeckers flap their wings more quickly than Pileated Woodpeckers is therefore counterintuitive. Further comment is conjecture: while the flight pattern and wing posture of the bird in the Luneau video may be unusual, it has not been shown that it is outside the range of variability of Pileated Woodpecker, and cannot therefore be used to eliminate the possibility that it was the commoner species.

**6. **'White plumage on the back is visible on the retreating bird as it begins to gain altitude. Ivory-billed Woodpecker has white on the back; Pileated Woodpecker has entirely black back.'

This was discussed by Sibley *et al *[4], who argued that the images thought to show white on the dorsum were too small to be accepted uncritically. In all the frames of the Luneau video that appear to show white on the dorsum, the bird is distant (dorsal white is not visible on the higher resolution images earlier in the video) and partially obscured, making it difficult to distinguish dorsum from wing. Spurious areas of white pixels appear as artifacts in both videos. Nevertheless, this remains the best evidence that the Luneau bird was not a standard Pileated Woodpecker.

7. 'The dorsal view of the right wing as it begins to unfold shows a triangle of white that matches in size and position the white on the folded wing of an Ivory-billed Woodpecker beginning to launch into flight.'

No further comment is provided here. An alternative explanation was offered by Sibley *et al *[4] and rebutted by Fitzpatrick *et al *[5]. The statement requires a degree of certainty about the position of the wing.

(8) 'The distance between the wrist area and the tip of the tail (32–36 cm, as measured when the bird begins to take flight) is comparable to known measurements of Ivory-billed Woodpecker and considerably larger than even the largest Pileated Woodpecker we measured.'

As stated under (3), above, there is general agreement that accurate measurements are not possible from the Luneau video because too many uncontrolled variables are involved [4,5].

9. 'Only 20 seconds before the woodpecker flees, a bird with the size and color pattern of an Ivory-billed Woodpecker was perched within 3 m of the site from which the woodpecker took flight.'

This would be a strong argument if it could be shown that the object in question was a bird and not, as is now apparently thought likely, a section of branch or tree stump [10]. The Luneau video reveals several white triangular patches apparently visible on or around tree trunks, most or all of which must therefore be images of tree topography or video artifacts. This was discussed in the literature (see [4,5]).

Central to the identification of the flying bird seen in the Luneau video was the evidence that plumage and flight patterns were inconsistent with Pileated Woodpecker. A very basic video analysis presented here has suggested that this may not be the case, and that further research is needed. Any identification of the bird in the Luneau video as an Ivory-bill must take into account the data presented here and in Sibley *et al *[4], which shows it is largely consistent with Pileated Woodpecker and points out apparent inconsistencies with Ivory-billed Woodpecker. This does not of course necessarily imply that the Ivory-billed Woodpecker is extinct, nor indeed entirely rule out the possibility that the bird in the Luneau video was one. There appears to be no reason to question the anecdotal sight records of Ivory-billed Woodpecker presented in Fitzpatrick *et al *[1] (or in many online sources), because some of them appear credible, albeit brief. Audio evidence has since been published [11] although this too is far from conclusive. However, to regard the Luneau video by itself as presenting anything other than an unidentified woodpecker falls below the standards of proof normally required for scientific publication: the images are not good enough. The Ivory-billed Woodpecker may persist in continental North America, and there is enough anecdotal evidence to make this a possibility, but the Luneau video does not support the case. The balance of evidence would suggest that the bird in the Luneau video is more likely to have been a Pileated Woodpecker, but the search for Ivory-billed Woodpecker should continue.

While this paper was under review, a report of sight records and sound recordings of Ivory-billed Woodpeckers was published from a location in Florida [12]. This very exciting claim is strengthened by reports of sighting of the white dorsal stripes on one bird in flight. Unfortunately, several sightings were made without optical aids and cannot be considered proven. The 'kent' calls recorded from the Florida location are spectrographically similar to the 'bleat' calls of young White-tailed Deer, as described in Richardson *et al *[13]. A clear photograph will be required from this location too before the presence of Ivory-billed Woodpeckers can be considered confirmed. It is hoped that this paper will help with assessment of any further low quality photographs or videos.

## Conclusion

Flight and plumage patterns of the putative Ivory-billed Woodpecker recorded in Arkansas in 2005 are recapitulated by confirmed Pileated Woodpeckers. The bird in the Arkansas video is best regarded as not fully identified, and is probably a Pileated Woodpecker.

## Methods

### Video recording

Pileated Woodpeckers were attracted to a bird feeder containing suet at Grants Trail, Dayton, OH 45459. The suet feeder was placed approximately 2.1 m high on a tree trunk, and the distance to the suet feeder from the observation point was approximately 5 m. Birds on the feeder were startled by movement of window blinds on January 28 and February 5, 2006, and their escape flights were filmed using a Sony Hi-8 SteadyShot video camera at 29.97 frames s^-1^. At least two birds feature in the videos, male and female.

Analogue tape was converted to digital by connecting the Hi-8 camera directly to a Sony DCR-HC30 digital video camera and recording onto that camera's mini dv cassette. The resulting images were converted to an avi file using Windows Movie Maker on a Windows XP PC. The video is freely available in wmv format [14] and in avi format from the author or David Nolin (via the author). The video was decompiled using Blaze Media Pro (Mystik Media, Hampstead, NC, USA) for a detailed analysis. Import into Avid^® ^Xpress Pro HD for deinterlacing did not reduce the wing flicker seen in the images, and further professional processing could not improve the resolution, so the original decompiled file was used for analysis. Hence some frames contain two overlaid images, which may lower the resolution in some cases. The decompiled file was examined frame by frame and compared to the decompiled images of the putative Ivory-billed Woodpecker presented in Fitzpatrick *et al *[1].

Wingbeat frequencies were calculated by noting the frame number of the midpoint of the downstroke of each wingbeat (e.g. Figure 1B, frame 758) and calculating the length of time taken per wingbeat as (number of frames between downstroke midpoints)/29.97.

## Authors' contributions

JMC performed the data analysis and drafted the manuscript.

## Supplementary Material

Additional file 1 Comparative frames from the Luneau video (A) and the four recorded Pileated Woodpecker escape flights (B-E) (.jpg image file). (A) Six wingbeats of the Luneau Woodpecker. (B) Three wingbeats of known Pileated Woodpecker. (C) Three wingbeats of known Pileated Woodpecker. (D) Seven wingbeats of known Pileated Woodpecker. (E) Nine wingbeats of known Pileated Woodpecker. Each row represents one wingbeat cycle. In each case, the left columns represent approximate midpoints of the downstroke, middle columns the approximate bottom of the downstroke, and right columns towards the top of the upstroke. There is evidence of a black trailing edge to the wing in frames 250, 366.7, possibly 466.7, 583.3 and 700 of the Luneau video (frames with both wings raised). The Pileated Woodpeckers are closer to the camera (and are at higher resolution) in comparable frames of B-E, and the black trailing wing edge is usually visible, but as the birds move away from the camera it becomes less obvious and, because of flexion of the wings, becomes interpretable (erroneously) as a black leading edge to the forewing, with a white trailing edge or (in middle columns) as broad 'wrap around' black wing-tips. Reproduced from [1] with permission from David Luneau. Click here for file

## Figures and Tables

**Table 1:** Frame numbers of David Nolin's video that mark the midpoint (wings approximately horizontal) of escape flight of Pileated Woodpeckers

	**Wingbeat no.**											
	**0**	**1**	**2**	**3**	**4**	**5**	**6**	**7**	**8**	**9**	**10**	**11**

**PIWO flight 1**	165	169	173	177	182	193	211	-	-	-	-	-
**PIWO flight 2**	303	307	310.5	316	321	327	334	341	348	358	364	369
**PIWO flight 3**	429	432.5	436	439.5	443	450	456	463	473	-	-	-
**PIWO flight 4**	747	751	754	758	762	767	771	777	783	790	797	-

**Table 2:** Mean wingbeat rates for escape flights of Pileated Woodpeckers over the first four wingbeats, the second four wingbeats, and the overall mean, combining both phases of flight

	**mean wingbeat rate (beats s**^-1^**)**		
	Wingbeats	Wingbeats	Overall

	1–4	5–8	mean

**PIWO flight 1**	7.1	-	3.9
**PIWO flight 2**	6.7	4.4	5.0
**PIWO flight 3**	8.6	4.0	6.7
**PIWO flight 4**	8.0	5.7	6.0
**Luneau video**	8.6	8.6	8.6

The underlined results represent rates that are comparable or equivalent to the woodpecker described in Fitzpatrick *et al *[1]. Calculations based on data presented in Table 1.
