# Supplementary figures and images for: Video analysis of the escape flight of Pileated Woodpecker Dryocopus pileatus: does the Ivory-billed Woodpecker Campephilus principalis persist in continental North America?
Source: BMC Biol. 2007 Mar 15;5:8. doi: 10.1186/1741-7007-5-8 (PMC1838407; doi:10.1186/1741-7007-5-8)

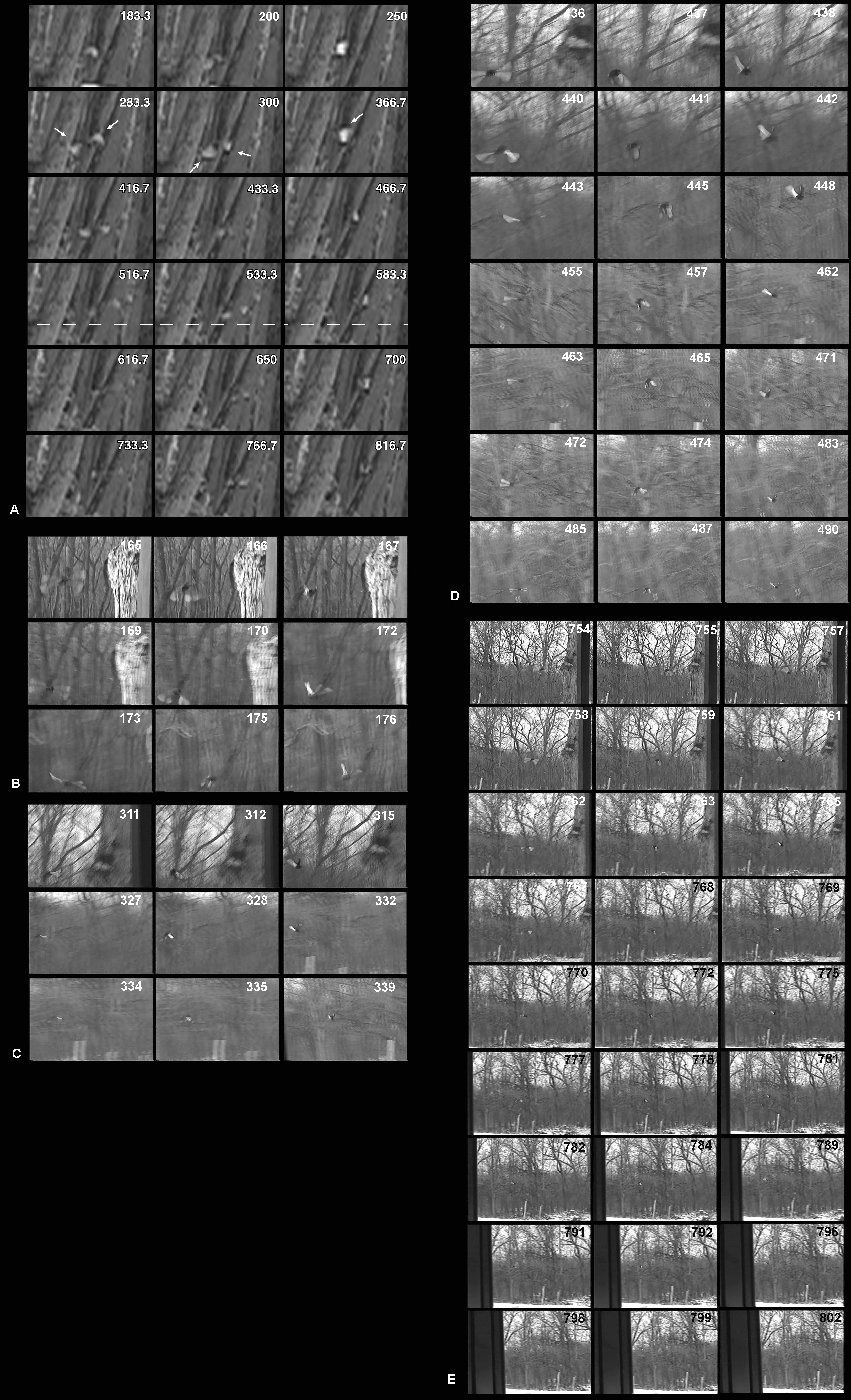

Supplement: Additional file 1 — Comparative frames from the Luneau video (A) and the four recorded Pileated Woodpecker escape flights (B-E) (.jpg image file). (A) Six wingbeats of the Luneau Woodpecker. (B) Three wingbeats of known Pileated Woodpecker. (C) Three wingbeats of known Pileated Woodpecker. (D) Seven wingbeats of known Pileated Woodpecker. (E) Nine wingbeats of known Pileated Woodpecker. Each row represents one wingbeat cycle. In each case, the left columns represent approximate midpoints of the downstroke, middle columns the approximate bottom of the downstroke, and right columns towards the top of the upstroke. There is evidence of a black trailing edge to the wing in frames 250, 366.7, possibly 466.7, 583.3 and 700 of the Luneau video (frames with both wings raised). The Pileated Woodpeckers are closer to the camera (and are at higher resolution) in comparable frames of B-E, and the black trailing wing edge is usually visible, but as the birds move away from the camera it becomes less obvious and, because of flexion of the wings, becomes interpretable (erroneously) as a black leading edge to the forewing, with a white trailing edge or (in middle columns) as broad 'wrap around' black wing-tips. Reproduced from [1] with permission from David Luneau. [file 1741-7007-5-8-S1.jpeg]
